# The Growth of the Nursing Profession: VIII. The Deeper and Wider Wants of the Nurse

**Published:** 1918-04-27

**Authors:** 


### THE MATRONS' AND SISTERS' DEPARTMENT.

### THE GROWTH OF THE NURSING PROFESSION.

### VIII.—The Deeper and Wider Wants of the Nurse.

One of the declared objects of the College of Nursing is to establish a central building which shall be the outward embodiment of the ideas for which the College stands. It is possible that at present this particular object, while universally approved, does not make an irresistible appeal to those nurses whose work lies outside London. But, to our thinking, this seemingly rather bald statement is symbolic of something far deeper and wider, which is of the utmost importance to future generations of nurses. It is the undeniable right of every individual to be surrounded by beauty as he or she understands it and is able to attain it. Even the sweated factory girl, earning but small wages each week, will give up her supper in order to satisfy that instinctive craving for life and beauty which in her uneducated consciousness manifests itself in the picture-house or a new hat. The main portion of a nurse's daily life is spent in the wards of a hospital or in sick-rooms, and she can never share that priceless blessing denied to all workers in institutions—her evenings and her Sundays at home. Hospital wards and private sick-rooms are often very bright and cheerful, and most pleasant places to work in, but sickness is not, was never meant to be, and never can be either natural or beautiful. When the nurse is off duty it should lie in her power to be in contact with the wholeness of life, a wholeness which must essentially result in beauty. And she should be able to do this easily and readily, without having to pull herself together and make a tremendous effort. She will naturally take the form of escape from hospital surroundings which is the nearest to her hand, and it is not always an inherent frivolity which leads many of our London nurses to find an intense pleasure in walking along Regent Street or Oxford Street just to look at the shops. Sometimes the readiest escape is a book, sometimes a church or a picture gallery, sometimes a 'bus ride to the nearest park, and frequently a picture-house or a tea-shop.

Now if the College, when it comes to design its central building, can eliminate from its mental vision every hospital or institution that ever was erected, and realise that it is building not for nurses as such, but for women who, whatever their different tastes, have a natural instinct for the beauty which is truth and the truth which is beauty, it will have accomplished much. Further, in every town where there is a centre, the College building, both externally and internally, should minister to the highest intellectual and artistic instincts of its members. By this we do not for one moment imply that the College should aim at an extrinsic beauty attractive only to the æsthete; very far from it. The primary function of such a building will no doubt be strictly professional, and it is professional libraries, professional help, and professional advantages that must first be provided. But if the building is to embody not merely the material aims for which the College stands, but also the noble ideals of life which should be the basis of all true nursing, it will have to co-ordinate professional activities with other manifestations of life equally essential to its wholeness. For instance, it is often said that nurses inevitably grow narrow-minded; and have little interest in general affairs or world-wide movements. No nurse under present conditions can be reasonably expected in her scant leisure to go out of her way to be interested in anything which does not directly affect her daily life. But we are convinced that the importance of our social and international problems of to-day is so great, that to a profession counting among its members so many of the women of our land, some incentive and assistance should be given in obtaining reliable information and a sufficiently wide basis for independent and thoughtful judgment.

There is nothing which involves more danger to the human fabric of our universe than one-sided development. Neither a life bound up in the four walls of hospital or institution or the small affairs of patients, nor a weak sentimentalism which enthuses over insipid attractions, whether in human beings, in books, in art, or in amusement, nor yet a violent partisanship in social or political affairs, which, being based on ignorance or half-knowledge, can do incalculable damage when it is not merely laughable (all of which types we have personally met in the profession)—none of these are desirable for the nurse. For this reason we voice a plea that every building bearing the name of the College of Nursing shall be of such generous scope both literally and figuratively as to attract the steps of those nurses who, escaping from the insistence of distorted daily life, are, whether consciously or unconsciously, seeking something which shall enable them once more to "see life steadily and see it whole."

